# Left Atrial Electrophysiological Properties after Pulmonary Vein Isolation Predict the Recurrence of Atrial Fibrillation: A Cohort Study

**DOI:** 10.31083/j.rcm2505167

**Published:** 2024-05-13

**Authors:** Yunfei Gu, Hao Wang, Guohua Xue, Yubing Guo, Pengyu Wu, Jingchao He, Aolin Ding, Songsen Li, Gary Tse, Tong Liu

**Affiliations:** ^1^Tianjin Key Laboratory of Ionic-Molecular Function of Cardiovascular Disease, Department of Cardiology, Tianjin Institute of Cardiology, The Second Hospital of Tianjin Medical University, 300211 Tianjin, China; ^2^Department of Cardiology, Luoyang Central Hospital Affiliated to Zhengzhou University, 471000 Luoyang, Henan, China; ^3^Biosense Webster Medical Technology, 471000 Luoyang, Henan, China

**Keywords:** atrial fibrillation, low voltage area, left atrial conduction time, recurrence

## Abstract

**Background:**

The aim of this work was to investigate left atrial electrophysiological properties for their ability to predict the recurrence of atrial fibrillation (AF) following pulmonary vein isolation (PVI).

**Methods:**

The study comprised 53 patients with AF [62 (interquartile range (IQR): 52–68) years old; 47.2% females]. High-density, three-dimensional electro-anatomic mapping using PentaRay was conducted during sinus rhythm in the left atrium (LA) immediately after PVI. LA conduction time, conduction velocity in predefined anterior and posterior routes, low voltage area percentage and distribution were assessed.

**Results:**

The AF recurrence group had longer LA conduction time compared to the non-recurrence group [11 (IQR: 10–12) ms vs. 9 (IQR: 8–10) ms, *p* = 0.001). The percent low voltage area was greater in the recurrence group than the non-recurrence group [31.2 (IRQ: 7.1–49.3)% vs. 7.7 (IQR: 4.3–15.2)%, *p* = 0.008]. Multivariate Cox regression revealed that LA conduction time independently predicted AF recurrence following ablation over a median follow-up of 235 days [IQR: 154–382 days; hazard ratio (HR): 2.37, 95% confidence interval (CI): 1.08–5.23, *p* = 0.031]. The optimal cut-off for LA conduction time was 98 ms [area under curve (AUC): 0.926, sensitivity: 0.833, specificity: 0.894, *p* < 0.01]. Kaplan–Meier analysis revealed that patients with a conduction time >98 ms had a higher rate of AF recurrence following ablation (*p* < 0.001).

**Conclusions:**

Patients with longer LA conduction time after PVI had more frequent AF recurrence.

## 1. Introduction

Atrial fibrillation (AF) is the most frequent arrhythmia found in aging societies [1, 2]. Its high incidence and prevalence are associated with greater risks of heart failure, stroke, dementia and death, thus making it a serious global public health issue [3, 4]. Currently, the initial recommended treatment for symptomatic patients with non-valvular and drug-refractory AF is pulmonary vein isolation (PVI) [5]. However, the post-ablation recurrence of AF remains an unsolved issue, and is reported to be about 10–50% over the first year [6, 7]. Atrial substrate fibrosis evaluated by atrial electrophysiological properties has been linked to the recurrence of AF [8, 9]. With recent development of intracardiac mapping technology, atrial endocardium bioelectric information can be obtained more directly and accurately [10]. The studies to date have mainly focused on parameters mapped before ablation [11, 12]. However, the electrical characteristics of the left atrium (LA) are reported to change greatly after pulmonary vein electrical isolation compared to before the procedure [13]. In the present study, we investigated whether the electrophysiological characteristics of the LA immediately after PVI are predictive of AF recurrence.

## 2. Methods

### 2.1 Study Cohort

This study included consecutive patients with AF who underwent PVI alone in the cardiology department of our hospital during September 2021 to July 2022. Inclusion criteria: (i) >18-years old; (ii) diagnosed with paroxysmal or persistent AF, as defined by European Society of Cardiology (ESC)/European Association for Cardio-Thoracic Surgery (EACTS) guidelines (2020) [3]; (iii) first-time treatment for PVI; (iv) gave fully informed consent. Exclusion criteria: (i) prior LA ablation, or prior cardiac surgery; (ii) patient cannot maintain sinus rhythm during voltage mapping, or additional ablations other than PVI; (iii) malignant tumor or serious disease of brain, liver, kidney or other major organ, with life expectancy of <1 year; (iv) pregnant or lactating. The study design and content received approval from the medical ethics committee of our institution. All data were analyzed anonymously and the individuals in this research provided written informed consent and participated voluntarily in the study.

### 2.2 Radiofrequency Catheter Ablation (RFCA)

Prior to RFCA, anti-arrhythmic drugs were stopped for more than five half-lives, but oral anticoagulation therapy continued. RFCA was performed under conscious sedation with fentanyl. CARTO®3 (version 6.0, Biosense Webster, Irvine, CA, USA) system was employed in this study. A 10-polar mapping catheter was inserted in the coronary sinus through the right internal jugular vein under X-ray guidance. After successful trans-septal puncture, heparin was injected at the appropriate dose to keep the activated clotting time (ACT) to 300–350 s. The Swartz sheath (L1 type, St. Jude Medical, St. Paul, MN, USA) was placed in the LA. Fast anatomic modeling (FAM) of the LA and bilateral pulmonary veins was conducted with a PentaRay® catheter (Biosense Webster, Irvine, CA, USA) followed by Thermo Cool SmartTouch® catheter (Biosense Webster, Irvine, CA, USA). Respiration gated training was conducted by inserting a PentaRay® catheter (Biosense Webster, Irvine, CA, USA) into the left inferior pulmonary vein. Circumferential pulmonary vein isolation (CPVI) was then carried out with a THERMOCOOL SMARTTOUCH® catheter (Biosense Webster, Irvine, CA, USA) at a power output of 35W and maximum temperature of 43 °C. The generation of ablation tags depends on the VisTag™ module (Biosense Webster, CA, USA), which contains two parameters: the stability max-range is set to 3 mm and to 3 s. Sufficient catheter–tissue contact was demonstrated by contact force sensing (5–20 g). The irrigation rate was 20 mL/min. Targets for the ablation index were 450–500 for the anterior wall, 350–400 for the posterior wall, and 450–550 for the LA roof. PVI was completed once both the entrance and exit blocks had been created.

### 2.3 Left Atrial Electrophysiological Properties Measurement

**Fig. 1.**
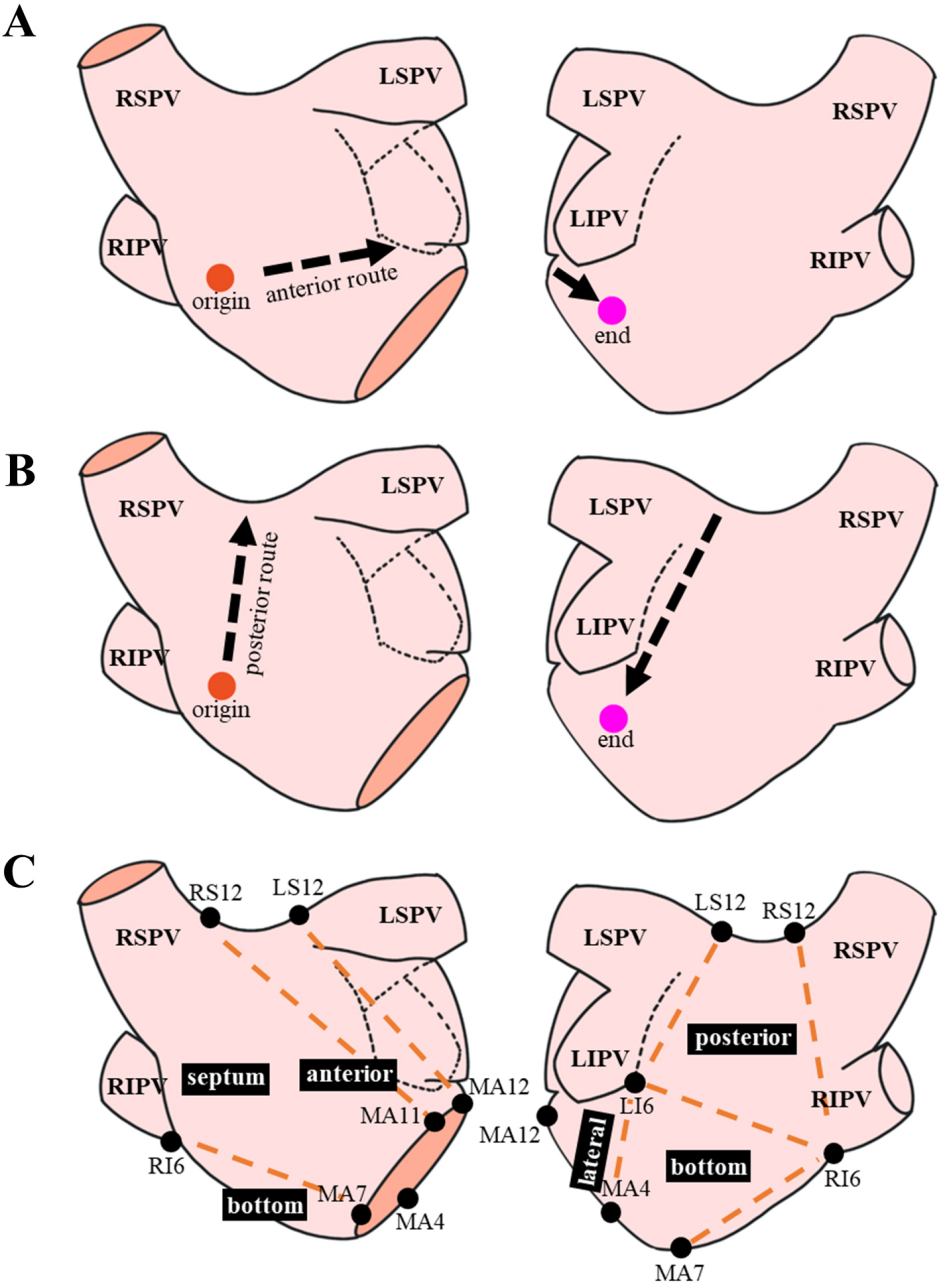
**Measurement of left atrial conduction routes and low voltage zone distributions**. (A,B) presented the anterior and posterior routes of LA conduction, respectively. The definition of LA conduction routes was as reported previously by Kurata *et al*. [14]. All anterior and posterior routes originated at the LA septum (red dots) and ended at the lateral mitral annulus (purple dots). They went along the anterior wall-appendage-LA appendage orifice (A) and the superior LA (roof)-posterior wall-left inferior pulmonary vein routes (B), respectively [14]. (C) showed the regional classification of LA, as described previously by Huo *et al*. [15]. Landmarks (black dots): MA12 (MA at 12 o’clock); MA4 (MA at 4); MA7 (MA at 7); MA11 (MA at 11); LS12 (LSPV at 12); RS12 (RSPV at 12); LI6 (LIPV at 6); RI6 (RIPV at 6). Surface regions were defined by: anterior wall, the area around LS12, RS12, MA12 and MA11; septum, the area around RI6, RS12, MA11 and MA7; bottom, the area around MA7, MA4, LI6 and RI6; lateral wall, the area around MA4, MA12, LI6 and LS12; posterior, the area around LI6, RI6, LS12 and RS12 [15]. Abbreviations: RSPV, right superior pulmonary vein; RIPV, right inferior pulmonary vein; LSPV, left superior pulmonary vein; LIPV, left inferior pulmonary vein; MA, mitral isthmus; LA, left atrium.

Mapping was conducted immediately after PVI using the PentaRay® catheter (Biosense Webster, Irvine, CA, USA) and at a 0.5 cm distance from the PVI ablation circle to avoid interference. LA conduction time was measured as the start to end of the propagation wave front in LA. Conduction distance was measured as reported previously [14], and included both the anterior and posterior routes (Fig. 1A,B, Ref. [14, 15]). LA conduction velocity (LACV) is defined as conduction distance divided by LA conduction time (described above). Since measurements were carried out manually, discrepancies may occur between repeat measurements. To reduce variability, inter-observer correlation coefficients of conduction distance were conducted in 10 randomly selected patients from the enrolled population using a two-way random-effects model. Bipolar voltage was measured in pairs with an inter electrode space of 2 mm for maximizing the accuracy of voltage data. Peak-to-peak signals were filtered at 16–500 Hz. To ensure sufficient point density, the minimum target for the endocardial voltage maps was 750 points, with a maximal density set to 1 mm. A low voltage area (LVA) had bipolar peak-to-peak voltage of <0.50 mV. A CARTO “area measurement” tool was used to delimitate the LVA extension. This tool allows low voltage extension to be drawn manually based on the color code of the selected threshold. The percent LVA in relation to total LA area was also calculated. LA was classified into 5 regions as described by Huo *et al*. [15]: anterior wall, septal, posterior wall, bottom, and lateral wall (Fig. 1C).

### 2.4 Follow-Up

Constant followed-up was conducted by well-trained and experienced staff through outpatient service, telephone or WeChat at 3- and 6-months after RFCA, then at 6-month intervals. In case of suspicious arrhythmia symptoms, 24-hour dynamic electrocardiogram monitoring or 30-day event monitoring was carried out according to the frequency of symptoms. AF recurrence was defined as symptomatic or asymptomatic AF, atrial tachycardia, or atrial flutter with a duration >30 s after the three-month blank period.

### 2.5 Statistical Analyses

Statistical analyses were performed with SPSS software (version 22.0; IBM SPSS Statistics, Chicago, IL, USA). Continuous variables are shown as the mean ± SD, and categorical variables as frequency and percentage. Significance tests for continuous variables were carried out with unpaired *t*-test or non-parametric tests (Mann-Whitney U test), and categorical variables with chi-square test or Fisher’s exact test. Univariable and multivariable Cox proportional hazards, together with Cox regression analysis was performed to analyze clinical features related to recurrence of AF. Kaplan–Meier analysis was employed to evaluate recurrence-free survival. Continuous variables that were found to correlate with AF recurrence were assessed by receiver operating characteristic (ROC) analysis. This identified the optimal cut-off value. The survival of groups defined by the optimal cut-off time was compared with the log-rank test. A *p* value of <0.05 was considered to represent statistical significance.

## 3. Results

### 3.1 Baseline Features of Participants

Of the 53 patients enrolled in this study, 6 suffered a recurrence of AF, giving a recurrence rate of 11.3%. Table 1 shows the patient baseline features for the recurrence and non-recurrence groups. There were no significant differences between these groups for patient age or gender, AF type and duration, comorbidity history, or any echocardiography parameters.

**Table 1.** **Baseline features of the study cohort**.

	Total	Recurrence	Non-Recurrence	*p* value
	(n = 53)	(n = 6)	(n = 47)	
Age (years)	62 (52, 68)	58 (51, 66)	62 (52, 69)	0.536
Female, n (%)	25 (47.2)	3 (50)	22 (46.8)	0.883
BMI (kg/$m^{2}$)	26.8 (24.6, 28.2)	27.1 (26.4, 28.4)	26.6 (24.4, 28.4)	0.482
Smoker, n (%)	13 (24.5)	2 (33.3)	11 (23.4)	0.595
Drinker, n (%)	6 (11.3)	2 (33.3)	4 (8.5)	0.071
Persistent AF, n (%)	20 (37.7)	2 (33.3)	18 (38.3)	0.813
AF duration (months)	12 (2, 48)	36 (18, 360)	12 (2, 48)	0.220
Hypertension, n (%)	32 (60.4)	5 (83.3)	27 (57.4)	0.222
DM, n (%)	11 (20.8)	1 (16.7)	10 (21.3)	0.793
CAD, n (%)	27 (50.9)	3 (50.0)	24 (51.1)	0.961
HF, n (%)	7 (13.2)	1 (16.7)	6 (12.8)	0.790
LAD (mm)	38 (35, 43)	40 (35, 45)	38 (35, 42)	0.510
LVEF (%)	65 (60, 70)	65 (60, 71)	65 (60, 68)	0.941

Abbreviations: BMI, body mass index; AF, atrial fibrillation; DM, diabetic mellitus; CAD, coronary artery disease; HF, heart failure; LAD, left atrial diameter; LVEF, left ventricular ejection fraction.

### 3.2 Procedural Parameters and Atrial Electrophysiological Properties

**Fig. 2.**
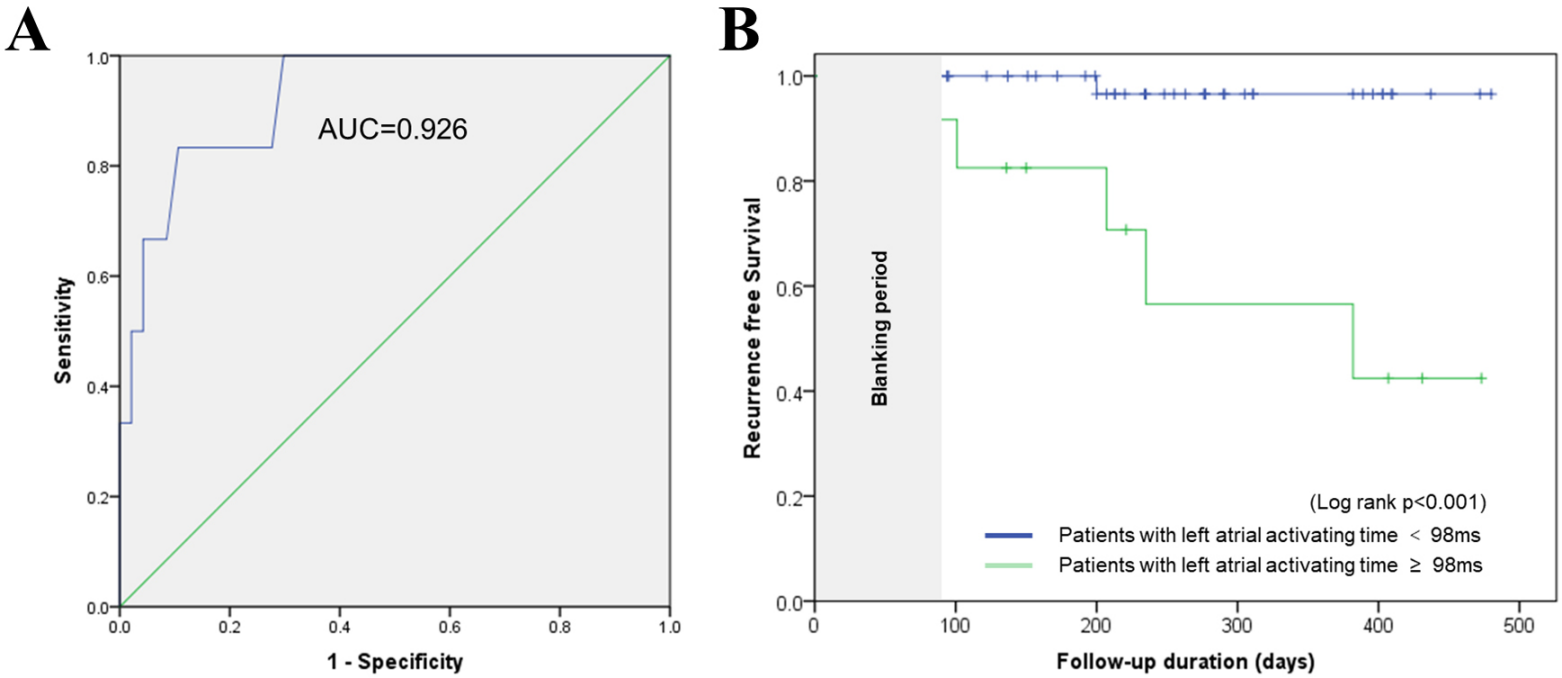
**Predictive value of post-PVI LA conduction time for AF recurrence**. (A) showed that the optimal cut-off for LA conduction time was 98 ms (AUC: 0.926, sensitivity: 0.833, specificity: 0.894, *p* < 0.01). (B) showed the Kaplan–Meier analysis result which revealed that patients with a conduction time >98 ms had a higher rate of AF recurrence following ablation (*p* < 0.001). Abbreviations: PVI, pulmonary vein isolation; LA, left atrium; AF, atrial fibrillation; AUC, area under curve.

No differences in the procedure or ablation times were observed between the two groups (Table 2). Atrial substrate mapping was conducted during sinus rhythm. Cardioversion was carried out to restore the sinus rhythm in AF cases following PVI. A 15-min pause in the mapping avoided any untoward effects due to the cardioversion. Seven patients accepted only one-time conversion after ablation, including two from the recurrence group (33.3%) and five from the non-recurrence group (10.6%), with no significant difference between them (Fisher’s exact test, *p* = 0.174). Table 2 shows the electrical properties of the LA. The two groups had similar numbers of mapping points [1240 (interquartile range (IQR): 1017–1289) vs. 967 (IQR: 840–1248), *p* = 0.159]. Inter-observer correlation coefficients for anterior and posterior route conducting distances in 10 random patients were respectively 0.954 [95% CI: 0.828–0.988, *p* < 0.001] and 0.986 [95% CI: 0.944–0.96, *p* < 0.001]. The LVA size (shown as a percentage) for the recurrence group was larger than the non-recurrence group [31.2 (IRQ: 7.1–49.3)% vs. 7.7 (IQR: 4.315.2)%, *p* = 0.008]. There was no significant difference in LVA distribution between the groups. Conduction time was longer in the recurrence than the non-recurrence group [108 (IQR: 97–122) ms vs. 85 (IQR: 75–95) ms, respectively, *p* < 0.001], while the conduction velocity for both the anterior and posterior routes was slower in the recurrence group [anterior route: 0.79 (IQR: 0.70–0.86) vs. 0.94 (IQR: 0.82–1.04), *p* = 0.020; posterior route: 0.87 (IQR: 0.75–1.03) vs. 1.08 (IQR: 0.96–1.22), *p* = 0.009; unit = m/s]. Table 3 shows the univariable and multivariable Cox regression analysis results for patient age and gender, body mass index (BMI), AF type and duration, left atrial diameter (LAD), LA volume (measured during ablation), LVA percentage, as well as LA conduction time (converted into 10 ms, given the clinical significance). LA conduction time was an independent predict of AF recurrence following PVI (HR: 2.37, 95% CI: 1.08–5.23, *p* = 0.031). The optimal cut-off value for LA conduction time, as determined by ROC curve analysis (Fig. 2A), was 98 ms (area under curve (AUC), 0.926; sensitivity, 0.833; specificity, 0.894, *p* < 0.01). Kaplan–Meier analysis showed worse AF recurrence-free survival in patients with a conduction time >98 ms compared to those with a conduction time ≤98 ms (*p* < 0.001, Fig. 2B). Fig. 2A shows the ROC curve of LA conduction time. At an optimal cut-off time of 98 ms, the AUC was 0.926, sensitivity was 0.833, and specificity was 0.894. Fig. 2B shows Kaplan-Meier analysis of AF recurrence-free survival in the two groups defined by the optimal cut-off of 98 ms for the LA conduction time (log-rank *p* < 0.001).

**Table 2.** **Comparison of procedural parameters and atrial electrophysiological properties**.

	Total	Recurrence	Non-Recurrence	*p* value
	(n = 53)	(n = 6)	(n = 47)	
Procedure time (min)	198 (156, 227)	185 (151, 266)	199 (155, 227)	0.455
Ablation time (min)	32 (28, 37)	32 (30, 34)	32 (28, 37)	0.820
Mapping points, n	1050 (841, 1253)	1240 (1017, 1289)	967 (840, 1248)	0.159
LA volume (mL)	87.0 (71.2, 106.1)	115.0 (76.7, 132.0)	82.8 (70.4, 96.7)	0.042
Low voltage area percentage (%)	7.8 (4.3, 17.0)	31.2 (7.1, 49.3)	7.7 (4.3, 15.2)	0.008
Low voltage zone distribution				
Anterior wall, n (%)	21 (39.6)	1 (16.7)	20 (42.6)	0.384
Septum, n (%)	37 (69.8)	3 (50.0)	34 (72.3)	0.351
Bottom, n (%)	19 (35.8)	3 (50.0)	16 (34.0)	0.655
Lateral, n (%)	13 (24.5)	2 (33.3)	11 (23.4)	0.627
Posterior wall, n (%)	15 (28.3)	2 (33.3)	13 (27.7)	0.998
LA conduction time (ms)	85 (76, 97)	108 (97, 122)	85 (75, 95)	<0.001
Anterior LACV (m/s)	0.90 (0.79, 1.02)	0.79 (0.70, 0.86)	0.94 (0.82, 1.04)	0.020
Posterior LACV (m/s)	1.07 (0.95, 1.20)	0.87 (0.75, 1.03)	1.08 (0.96, 1.22)	0.009

Abbreviations: LA, left atrium; LACV, left atrial conduction velocity.

**Table 3.** **Univariate and multivariate Cox regression analysis**.

Variable	Univariate		Multivariate	
	HR (95% CI)	*p* value	HR (95% CI)	*p* value
Age	0.99 (0.91–1.09)	0.912	1.03 (0.91–1.15)	0.664
Female	0.66 (0.13–3.30)	0.612	0.56 (0.08–3.85)	0.553
BMI	1.12 (0.85–1.48)	0.419		
Persistent AF	0.68 (0.12–3.70)	0.651		
AF duration	1.01 (0.99–1.03)	0.158		
LA diameter	1.05 (0.91–1.22)	0.498		
LA volume	1.03 (1.00–1.06)	0.050		
LVA percentage	1.06 (1.01–1.10)	0.010	1.01 (0.95–1.07)	0.723
LA conduction time (10 ms)	2.33 (1.44–3.76)	0.001	2.37 (1.08–5.23)	0.031

Abbreviations: LVA, low voltage area; HR, hazard ratio; BMI, body mass index; AF, atrial fibrillation; LA, left atrium.

## 4. Discussion

This study found that longer LA conduction time measured immediately after PVI independently predicted AF recurrence after ablation, with the optimal cut-off time being 98 ms.

Moreover, LA conduction time after PVI was closely related to the recurrence of AF. Every 10 ms increase in LA conduction time increased the recurrence 1.37-fold. LA conduction time is associated with the conduction velocity of LA myocytes, as well as the size of the LA. These reflect the electrolytic and structural reconstruction of the atria, respectively. Electrical remodeling in AF patients may be detected before structural remodeling [16], and manifests mostly as a shortened atrial effective refractory period, maladaptation to changes in heart rate, and prolongation of the intra-atrial conduction time (IACT). *In vitro* research suggests the changes in electrical properties of the atrial myocytes may be related to intracellular calcium dynamics [17]. Atrial oxygen consumption increased during AF, resulting in the depletion of intracellular adenosine triphosphate (ATP). Along with the reduction in ATP level, calcium failed to be removed from the intracellular environment, leading to calcium overload. The ATP-dependent potassium channel then opens, resulting in a shortened duration of cardiac action potential and poor heart rate adaptation. Verapamil can reverse this change, thereby supporting this hypothesis [18].

In addition to electrical characteristics such as the depolarization ability of single cells, the conduction velocity also depends on the gap junction function between myocardial cells [19]. Gap junctions are closely packed channels connecting directly to the cytoplasm of adjacent cells, allowing passage for small molecules (<1 kDa) and ions [20]. Pharmacological agents that inhibit gap junctions are known to reduce the conduction velocity of action potentials that travel through working myocardium [21, 22]. Transgenic mice with targeted *Cx40* gene deletion have prolonged PQ interval and duration of P-wave and QRS complex wave [23, 24].

LA size is closely correlated to AF recurrence following surgery. This is usually measured using parameters such as the anteroposterior LAD, LA volume, and volume index of the LA [obtained by echocardiography, computed tomography (CT), magnetic resonance imaging (MRI), or three-dimensional mapping]. A meta-analysis by Zhuang *et al*. [25] found the average LAD in patients suffering a recurrent AF was 1.84 mm larger than in those without recurrence [25]. Njoku *et al*. [26] reported the AF recurrence rate increased by 3% for each unit of increase in the LA volume index. Structural remodeling, such as cellular hypertrophy and increased tension during atrial enlargement, can promote the secretion of various cytokines including cardiac endothelin-1 and vascular endothelial growth factor. These subsequently recruit macrophages, thereby further promoting cardiac cell hypertrophy and interstitial fibrosis. Atrial structural changes secondary to AF, such as cell loss caused by degeneration, fibrosis and apoptosis, lead to the depletion of gap junctions and to their abnormal distribution [27, 28, 29, 30]. The increased myocardial cell gap interrupts the electrical connection between muscle bundles, which then induces dysfunctional tissue coupling, non-continuous propagation, and non-uniform anisotropic conduction resulting in intra-atrial conduction block [31]. These changes can induce the formation and maintenance of atrial micro-reentry. Moreover, although the precise relationship between atrial enlargement and the onset of AF remains unclear, LA enlargement is often an indicator of worse cardiac function and longer duration of AF, all of which contribute to an increased risk of recurrence.

Earlier studies examined the predictive value of atrial conduction time for AF recurrence, although this was usually measured before PVI [32, 33]. The muscular sleeve of the pulmonary vein constitutes the end part of the total conduction time of LA [34]. The mapped conduction time after PVI should therefore better reflect the electrophysiological state of atrial substrate. In baseline comparisons, the average conduction velocity of the LA along the anterior and posterior routes was different between recurrence and non-recurrence groups.

The conduction path in the LA is multi-origin, multi-time and multi-dimensional. For example, as mentioned in the methods section, Kurata *et al*. [14] divided the paths into anterior and posterior routes according to the start and end of the propagation wave front. Sato *et al*. [35] classified three pathways: roof, anterior, and septal. Ohguchi *et al*. [36] used a defined LACV model that was based on an orthogonal projection vector calculated within the triangle area. There is currently no standard measurement path, which makes it difficult to combine and compare data between different studies. In our multivariable Cox regression analysis, we chose to include LA conduction time instead of the conduction velocity, mainly for the following reasons. Firstly, the measurement of conduction time is intuitive. At the end of mapping, it can be obtained directly by comprehensively analyzing the information from all the mapping point. The conduction time therefore has high practical value in the clinic. Secondly, right atrial excitement spreads to LA through at least three breakthrough points: (i) anterior via the Bachmann bundle, (ii) posterior through myocardial pathways or bridges connecting atriums at the right pulmonary vein level (also referred to as fossa ovalis connections), (iii) inferior through myocardial sleeves that extend from coronary sinus ostium and coronary sinus musculature to inferior portions of LA wall.

In the present study, LVA was not independently related to post-ablation AF recurrence. This may be due to the following reasons. First, the timing of LVA mapping was different. Previous research on LVA was focused mainly on pre-PVI mapping, either under AF rhythm or after cardioversion [11, 12]. LVA mapping itself can be affected by many factors. Bipolar voltage mapping (BVM) is the leading method for low voltage area mapping, but can be affected by the direction of the propagation wave front. The speed of conduction can also affect arrival times of the propagation wave front at each electrode, thus changing bipolar signal amplitude and shape [37] in a phenomenon known as bipolar blindness. Chierchia *et al*. [38] found that LVA mapped with the in-sinus rhythm in AF patients, with only partial overlap during coronary sinus pacing. Compared with coronary sinus pacing, the average amplitude during sinus rhythm was significantly higher, indicating the directional dependence of amplitude of bipolar signal [38]. Additionally, factors such as electrode spacing and size, degree of tissue contact, signal filtering (references selected), and number of mapping points can also affect the mapping results for LVA [39]. These could explain some of the discordant results between studies. Moreover, there is no standardized cut-off value for LVA, meaning there would be different LVA sizes.

## 5. Limitations

This investigation has a number of limitations, including its single-center, non-random, and observational study design. The sample size was relatively modest. Bias is possible due to the selection of clinical variables and different atrial matrices between groups. To examine the reliability of our conclusions, PASS software (Power Analysis and Sample Size Software (2021) NCSS, LLC Kaysville, UT, USA, https://ncss.com/software/pass) was used to calculate the statistical power using the following parameters: sample size (N) = 53, reg. coef. (B) = 0.8629 (the ln transformation for a HR of 2.37), event rate = 11.3% (6/53), and two-sided α = 0.05. A power of 88.64% was found for the current sample size, which is within the acceptable range of statistical power [40, 41]. The original output document from the PASS software analysis is shown in **Supplementary File 1**. In addition, we also conducted a meta-analysis, which finally found that the LA conduction time in the recurrence group was about 15 ms longer than in the non-recurrence group, with a 95% CI of 8.9 ms to 21.7 ms (*p* < 0.001) (**Supplementary File 2**). Moreover, the LVA percentage was not significantly different between the two groups (mean difference: 4.54%, 95% CI: –1.11%–10.18%, *p* = 0.12), consistent with the present results. Despite these compensations, subgroup analysis was not conducted because of the small sample size, which is another limitation of this study. Thirdly, the ablation of AF was time-consuming. In view of patient tolerance, only the LA was mapped. Finally, several other possible predictors of AF clinical recurrence such as serum NT-proBNP levels and LA reservoir strain analysis [42, 43] were not included in the current study. These should be explored further in multi-centered, prospective studies.

## 6. Conclusions

Longer LA conduction time after PVI increased the risk of AF recurrence.

## Data Availability

The datasets used and analyzed during the current study are available from the corresponding authors on reasonable request.

## Supplementary Material

Supplementary material associated with this article can be found, in the online version, at https://doi.org/10.31083/j.rcm2505167.
