# Supplementary material for: Left Atrial Electrophysiological Properties after Pulmonary Vein Isolation Predict the Recurrence of Atrial Fibrillation: A Cohort Study
Source: Rev Cardiovasc Med. 2024 May 13;25(5):167. doi: 10.31083/j.rcm2505167 (PMC11267206; doi:10.31083/j.rcm2505167)
Supplement: Supplementary file 1 [file 2153-8174-25-5-167-s1.pdf]

Cox Regression

Numeric Results

Power	Sample Size (N)	Reg. Coef. (B)	S.D. of X1 (SD)	Event Rate (P)	R-Squared X1 vs Other X's (R2)	Two-Sided Alpha	Beta
0.8864	53	0.8629	1.5	0.113	0	0.05	0.1136

References

Hsieh, F.Y. and Lavori, P.W. 2000. 'Sample-Size Calculations for the Cox Proportional Hazards Regression Model with Nonbinary Covariates', Controlled Clinical Trials, Volume 21, pages 552-560.
Schoenfeld, David A. 1983. 'Sample-Size Formula for the Proportional-Hazards Regression Model', Biometrics, Volume 39, pages 499-503.

Report Definitions

Power is the probability of rejecting a false null hypothesis. It should be close to one.
N is the size of the sample drawn from the population.
B is the size of the regression coefficent to be detected
SD is the standard deviation of X1.
P is the event rate.
R2 is the R-squared achieved when X1 is regressed on the other covariates.
Alpha is the probability of rejecting a true null hypothesis.
Beta is the probability of accepting a false null hypothesis.

Summary Statements

A Cox regression of the log hazard ratio on a covariate with a standard deviation of 1.5 based on a sample of 53 observations achieves 89% power at a 0.05 significance level to detect a regression coefficient equal to 0.8629. The sample size was adjusted for an anticipated event rate of 0.113.

Dropout-Inflated Sample Size

Dropout Rate	Sample Size N	Dropout-Inflated Enrollment Sample Size N'	Expected Number of Dropouts D
20%	53	67	14

Definitions

Dropout Rate (DR) is the percentage of subjects (or items) that are expected to be lost at random during the course of the study and for whom no response data will be collected (i.e., will be treated as "missing").
N is the evaluable sample size at which power is computed (as entered by the user). If N subjects are evaluated out of the N' subjects that are enrolled in the study, the design will achieve the stated power.
N' is the total number of subjects that should be enrolled in the study in order to end up with N evaluable subjects, based on the assumed dropout rate. N' is calculated by inflating N using the formula N' = N / (1 - DR), with N' always rounded up. (See Julious, S.A. (2010) pages 52-53, or Chow, S.C., Shao, J., Wang, H., and Lokhnygina, Y. (2018) pages 32-33.)
D is the expected number of dropouts. D = N' - N.

Cox Regression

Procedure Input Settings

Autosaved Template File
C:\Users\Alexis\Documents\PASS 2021\Procedure Templates\Autosave\Cox Regression - Autosaved 2023_11_2-23_28_26.t161

Design Tab

Solve For:	Power
Alternative Hypothesis:	Two-Sided
Alpha:	0.05
N (Sample Size):	53
P (Overall Event Rate):	0.113
B (Log Hazard Ratio):	0.8629
R-Squared of X1 with Other X's:	0.0
S (Standard Deviation of X1):	1.5

The results achieved by meta-analysis mentioned above are shown in the following contents.

Study selection of the meta-analysis

All studies exploring the association between left atrial electronic properties and recurrence of AF after ablation published before January 31st 2023 were included. Studies were searched by searching the PubMed, EMBASE and SCOPUS database using different combinations of terms, including “conduction”, “atrial fibrillation” and “recurrence”. All studies retrieved in this search were reviewed by two independent researchers (YG and HW) in parallel to minimize subjective selection bias. Divergences were adjudicated by discussion with a third investigator (GX). Studies were excluded if they fulfilled the following criteria: (i) animal experiments and mechanistic studies; (ii) clinical studies but using cohort design; (iii) data were overlapping or unavailable. Only articles in published in English were included.

Statistical analysis

Meta-analysis was conducted using Review Manager (version 5.3).Heterogeneity was evaluated based on the I^2 metric of inconsistency and the Chi-squared-based Cochran Q test. The value of I^2 reflected the proportion of the impacts caused by between-study heterogeneity rather than sampling error. In the absence of notable heterogeneity ($I^2 < 50\%$), a fixed effect model was used to calculate the effect size; when heterogeneity was detected ($I^2 \geqslant 50\%$), a random-effects model was used. Detailed differences between the two models were described in our previous studies. The z-test was used to assess the combined statistical outcomes. A two tailed value of $p < 0.05$ was considered statistically significant.

Results of meta-analysis

A total of 1,533 potential studies were retrieved by the initial search. Among these studies, 2,815 were discarded by screening titles, abstracts, or full-length texts, as summarized in Figure S1. Ultimately, 3 studies and 2 study were respectively included in the final analysis of left atrial conduction time and LVA. As shown in Figures S2A and S2B, left atrial conduction time was about 15ms longer in recurrence group than non-recurrence group, with 95%CI of 8.9ms to 21.7ms ($p<0.001$). By contrast, the difference of LVA percentage between the two groups was not significant (mean difference: 4.54%, 95%CI: -1.11%-10.18%, p=0.12).

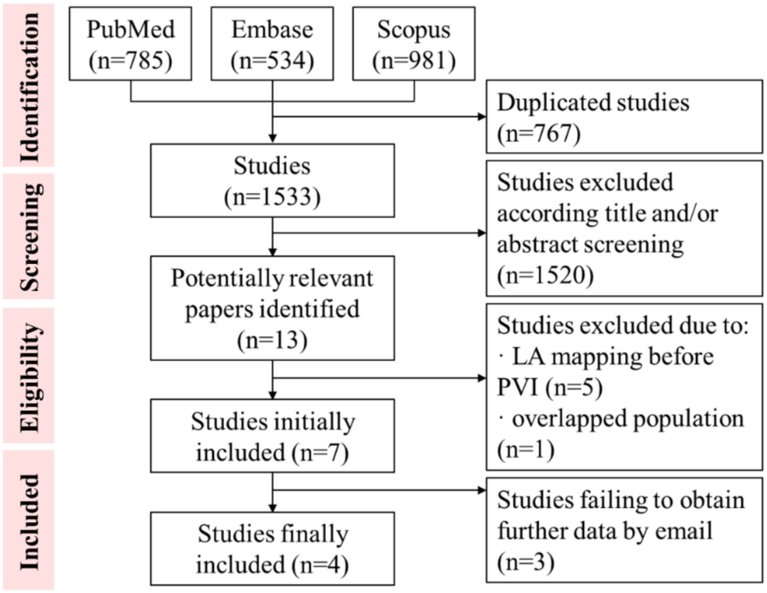

Figure S1 Flow chart of meta-analysis

A

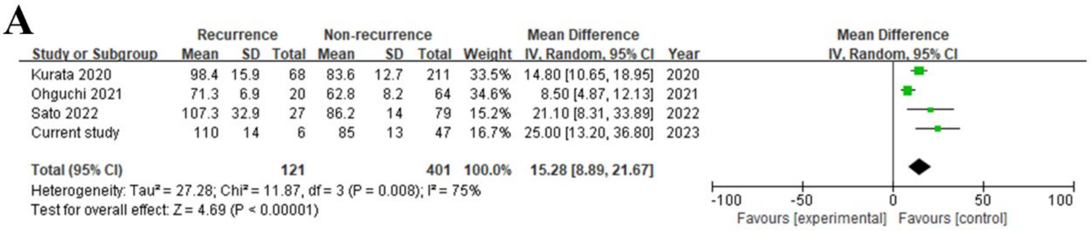

B

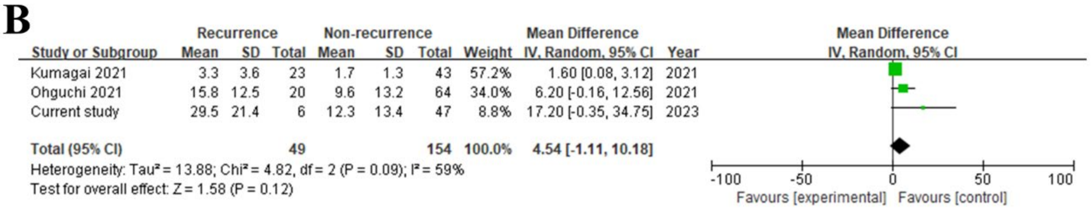

Figure S2 Forest plot showing the difference in left atrial electrophysiological properties between recurrence and non-recurrence patients after ablation.

with post-RFAAF recurrence had a higher mean Left Atrial volume compared to patients with no recurrence

The upper panel (A) showed the left atrial conduction time was longer in patients with AF recurrence (mean difference: 15.28ms, 95%CI: 8.89ms to 21.67ms, p < 0.001). Panel B showed that patients with post-procedure recurrence had similar low voltage area percentage with those without recurrence (mean difference: 4.54%, 95%CI: -1.11% to 10.18%, p=0.12).

(End of meta-analysis)
